# Hypertensive Disorders in Pregnancy and Mortality at Delivery Hospitalization — United States, 2017–2019

**DOI:** 10.15585/mmwr.mm7117a1

**Published:** 2022-04-29

**Authors:** Nicole D. Ford, Shanna Cox, Jean Y. Ko, Lijing Ouyang, Lisa Romero, Tiffany Colarusso, Cynthia D. Ferre, Charlan D. Kroelinger, Donald K. Hayes, Wanda D. Barfield

**Affiliations:** ^1^Division of Reproductive Health, National Center for Chronic Disease Prevention and Health Promotion, CDC; ^2^Division of Heart Disease and Stroke Prevention, National Center for Chronic Disease Prevention and Health Promotion, CDC.

Hypertensive disorders in pregnancy (HDPs), defined as prepregnancy (chronic) or pregnancy-associated hypertension, are common pregnancy complications in the United States.* HDPs are strongly associated with severe maternal complications, such as heart attack and stroke (*1*), and are a leading cause of pregnancy-related death in the United States.^†^ CDC analyzed nationally representative data from the National Inpatient Sample to calculate the annual prevalence of HDP among delivery hospitalizations and by maternal characteristics, and the percentage of in-hospital deaths with an HDP diagnosis code documented. During 2017–2019, the prevalence of HDP among delivery hospitalizations increased from 13.3% to 15.9%. The prevalence of pregnancy-associated hypertension increased from 10.8% in 2017 to 13.0% in 2019, while the prevalence of chronic hypertension increased from 2.0% to 2.3%. Prevalence of HDP was highest among delivery hospitalizations of non-Hispanic Black or African American (Black) women, non-Hispanic American Indian and Alaska Native (AI/AN) women, and women aged ≥35 years, residing in zip codes in the lowest median household income quartile, or delivering in hospitals in the South or the Midwest Census regions. Among deaths that occurred during delivery hospitalization, 31.6% had any HDP documented. Clinical guidance for reducing complications from HDP focuses on prompt identification and preventing progression to severe maternal complications through timely treatment (*1*). Recommendations for identifying and monitoring pregnant persons with hypertension include measuring blood pressure throughout pregnancy,^§^ including self-monitoring. Severe complications and mortality from HDP are preventable with equitable implementation of strategies to identify and monitor persons with HDP (*1*) and quality improvement initiatives to improve prompt treatment and increase awareness of urgent maternal warning signs (*2*).

Delivery hospitalization data for 2017–2019 were analyzed from the National Inpatient Sample, a nationally representative sample of all U.S. hospital discharges.^¶^ CDC identified delivery hospitalizations among females aged 12–55 years using *International Classification of Diseases, Tenth Revision, Clinical Modification* (ICD-10-CM) diagnosis and procedure codes pertaining to delivery and diagnosis-related group delivery codes.** HDPs were categorized using ICD-10-CM diagnosis codes^††^ for chronic hypertension,^§§^ pregnancy-associated hypertension,^¶¶^ and unspecified maternal hypertension. Deaths were identified based on patient hospital discharge disposition.

Weighted annual prevalence (percentage) and 95% CI for HDP overall and by each type were calculated. Change in annual prevalence of HDP overall and by type was assessed using a linear trend test. Pooling data from this period, CDC calculated the weighted prevalence and 95% CIs for HDP by selected maternal characteristics (i.e., age group, race and ethnicity, and primary payer at delivery hospitalization) and characteristics of the community in which they lived (i.e., county-level rural-urban classification, zip code–level median household income, and hospital region).*** Rao-Scott chi-square tests of independence were used to assess whether HDP prevalence differed by characteristics. Percentage of deaths during delivery hospitalization with a documented HDP diagnosis code were calculated. All analyses were conducted using SAS software (version 9.4; SAS Institute); SAS survey procedures and weighting were used to account for complex sampling in the National Inpatient Sample. This activity was reviewed by CDC and was conducted consistent with applicable federal law and CDC policy.^†††^

During 2017–2019, the prevalence of HDP among delivery hospitalizations increased from 13.3% to 15.9% (Figure 1), an increase of approximately 1 percentage point annually. Linear trend tests suggested that change in annual prevalence of HDP overall, pregnancy-associated hypertension, and chronic hypertension increased during 2017–2019, while prevalence of unspecified maternal hypertension remained stable. The prevalence of pregnancy-associated hypertension increased from 10.8% to 13.0% and that of chronic hypertension increased from 2.0% to 2.3%.

**FIGURE 1:**
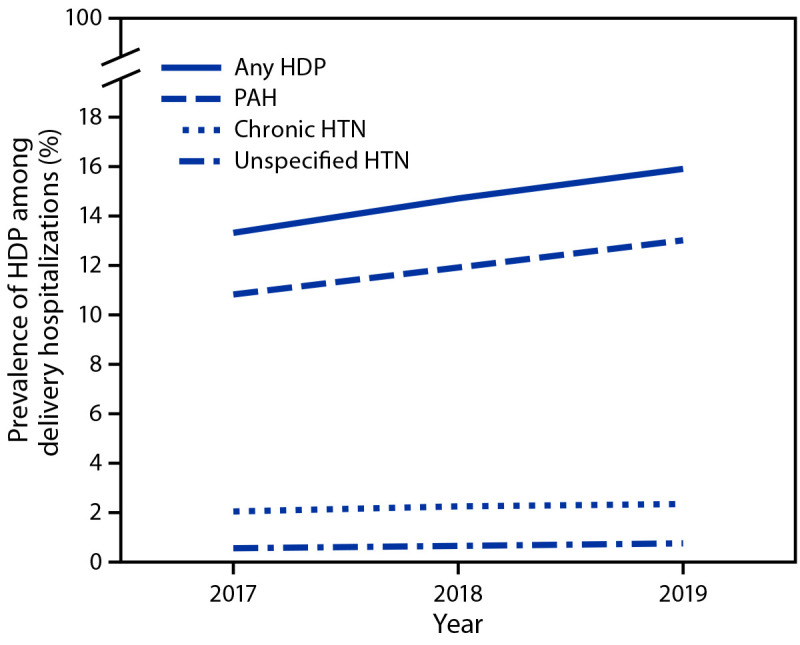
Prevalence of hypertensive disorders in pregnancy* among delivery hospitalizations, by year — National Inpatient Sample, United States, 2017–2019 **Abbreviations:** HDP = hypertensive disorder in pregnancy; HTN = hypertension; PAH = pregnancy-associated hypertension. * HDPs are defined as chronic hypertension, pregnancy-associated hypertension (i.e., gestational hypertension, preeclampsia, eclampsia, and chronic hypertension with superimposed preeclampsia), and unspecified maternal hypertension.

During 2017–2019 combined, prevalence of HDP overall was 14.6%. Prevalence varied overall and by HDP type for all maternal characteristics evaluated in the study (Table). Prevalence of any HDP was higher among delivery hospitalizations to women aged 35–44 (18.0%) and 45–55 years (31.0%) than to younger women, to Black (20.9%) and AI/AN (16.4%) women than to women of other racial and ethnic groups, to those residing in rural counties (15.5%) and in zip codes in the lowest median household-level income quartile (16.4%) than those residing in metropolitan or micropolitan counties or in zip codes in higher household-level income quartiles, or delivering in hospitals in the South (15.9%) or Midwest (15.0%) U.S. Census regions than in other Census regions. These differences in HDP prevalence were similar across HDP types.

**TABLE:** Prevalence of hypertensive disorders in pregnancy, by patient-, hospital- and zip code–level characteristics — National Inpatient Sample, United States, 2017–2019

Characteristic	Any hypertensive disorder in pregnancy*		Chronic hypertension		Pregnancy-associated hypertension		Unspecified maternal hypertension	
	No.^†^	Row % (95% CI)	No.	Row % (95% CI)	No.	Row % (95% CI)	No.	Row % (95% CI)
**Total no. of cases**	**319,913**	**—**	**47,218**	**—**	**259,458**	**—**	**13,237**	**—**
**Maternal age group, yrs**								
12–24	73,421	13.9 (13.7–14.1)	5,593	1.1 (1.0–1.1)	65,378	12.4 (12.2–12.5)	2,450	0.5 (0.4–0.5)
25–29	85,358	13.5 (13.3–13.7)	10,984	1.7 (1.7–1.8)	71,010	11.2 (11.1–11.4)	3,364	0.5 (0.5–0.6)
30–34	89,242	14.3 (14.1–14.4)	14,982	2.4 (2.3–2.4)	70,287	11.2 (11.1–11.4)	3,973	0.6 (0.6–0.7)
35–44	70,395	18.0 (17.7–18.2)	15,341	3.9 (3.8–4.0)	51,672	13.2 (13.0–13.4)	3,382	0.9 (0.8–0.9)
45–55	1,497	31.0 (29.7–32.4)	318	6.6 (5.9–7.3)	1,111	23.0 (21.8–24.2)	68	1.4 (1.1–1.7)
**Race and ethnicity** ^§^								
Asian or Pacific Islander	12,183	9.3 (8.8–9.7)	1,616	1.2 (1.1–1.3)	10,134	7.7 (7.3–8.1)	433	0.3 (0.3–0.4)
Black	66,316	20.9 (20.5–21.2)	13,639	4.3 (4.2–4.4)	49,568	15.6 (15.3–15.9)	3,109	1.0 (0.9–1.0)
Hispanic	54,702	12.5 (12.2–12.8)	6,561	1.5 (1.5–1.5)	46,148	10.6 (10.3–10.8)	1,993	0.5 (0.4–0.5)
American Indian and Alaska Native	2,525	16.4 (15.4–17.5)	318	2.1 (1.8–2.3)	2,103	13.7 (12.7–14.6)	104	0.7 (0.5–0.8)
Another race	11,659	12.0 (11.6–12.3)	1,400	1.4 (1.4–1.5)	9,781	10.1 (9.7–10.4)	478	0.5 (0.4–0.5)
White	162,122	14.7 (14.5–14.9)	22,358	2.0 (2.0–2.1)	133,052	12.1 (11.9–12.2)	6,712	0.6 (0.6–0.6)
Missing	10,406	12.7 (12.2–13.1)	1,326	1.6 (1.5–1.7)	8,672	10.6 (10.2–11.0)	408	0.5 (0.4–0.6)
**Payer**								
Public^¶^	139,227	14.8 (14.6–15.0)	21,541	2.3 (2.2–2.3)	111,543	11.8 (11.7–12.0)	6,143	0.7 (0.6–0.7)
Private insurance	166,455	14.8 (14.7–15.0)	23,826	2.1 (2.1–2.2)	136,153	12.1 (12.0–12.3)	6,476	0.6 (0.6–0.6)
Self-pay/Other	13,837	11.9 (11.6–12.2)	1,791	1.5 (1.5–1.6)	11,443	9.8 (9.5–10.1)	603	0.5 (0.5–0.6)
**Rurality (county-level)**								
Metropolitan	275,342	14.6 (14.4–14.8)	40,136	2.1 (2.1–2.2)	224,232	11.9 (11.7–12.0)	10,974	0.6 (0.6–0.6)
Micropolitan	25,844	14.8 (14.5–15.0)	4,026	2.3 (2.2–2.4)	20,497	11.7 (11.5–11.9)	1,321	0.8 (0.7–0.8)
Rural**	18,139	15.5 (15.1–15.8)	2,980	2.5 (2.4–2.7)	14,241	12.1 (11.9–12.4)	918	0.8 (0.7–0.8)
**Median household-level income national quartile for patient zip code** ^††^								
Q1	98,661	16.4 (16.1–16.6)	16,218	2.7 (2.6–2.8)	78,022	12.9 (12.7–13.2)	4,421	0.7 (0.7–0.8)
Q2	81,089	14.7 (14.5–14.9)	11,916	2.2 (2.1–2.2)	65,747	11.9 (11.8–12.1)	3,426	0.6 (0.6–0.6)
Q3	77,387	14.4 (14.3–14.6)	10,829	2.0 (2.0–2.1)	63,629	11.9 (11.7–12.0)	2,929	0.5 (0.5–0.6)
Q4	60,014	12.7 (12.5–12.9)	7,830	1.7 (1.6–1.7)	49,857	10.5 (10.3–10.7)	2,327	0.5 (0.5–0.5)
**Hospital region** ^§§^								
Northeast	48,527	13.9 (13.5–14.4)	6,746	1.9 (1.8–2.0)	40,017	11.5 (11.1–11.9)	1,764	0.5 (0.5–0.5)
Midwest	69,181	15.0 (14.7–15.3)	9,736	2.1 (2.0–2.2)	56,611	12.3 (12.0–12.5)	2,834	0.6 (0.6–0.6)
South	136,435	15.9 (15.7–16.2)	22,355	2.6 (2.5–2.7)	107,940	12.6 (12.4–12.8)	6,140	0.7 (0.7–0.7)
West	65,770	12.7 (12.4–13.0)	8,381	1.6 (1.6–1.7)	54,890	10.6 (10.4–10.9)	2,499	0.5 (0.5–0.5)

** Abbreviation:** Q = quartile.

* Any hypertensive disorder in pregnancy includes chronic hypertension, pregnancy-associated hypertension, and unspecified maternal hypertension.

^†^ Numbers are unweighted.

^§ ^Patients with Hispanic ethnicity are classified as Hispanic and all non-Hispanic patients are classified according to their reported race. The Healthcare Cost and Utilization Project (HCUP) race and ethnicity category Native American is expressed as American Indian and Alaska Native.

^¶^ Public insurance includes Medicare and Medicaid.

** Rural defined as nonmetropolitan and nonmicropolitan counties.

^††^ 2017 (Q1 = $1–$43,999, Q2 = $44,000–$55,999, Q3 = $56,000–73,999, Q4 = ≥$74,000); 2018 (Q1 = $1–$45,999, Q2 = $46,000–$58,999, Q3 = $59,000–$78,999, Q4 = ≥$79,000); 2019: Q1 = $1–$47,999, Q2 = $48,000–$60,999, Q3 = $61,000–$81,999, Q4 = ≥$82,000.

^§§^ Hospital region is the census region as defined by the U.S. Census Bureau.

Among maternal deaths that occurred during delivery hospitalization, 31.6% had any HDP documented and 24.3% had pregnancy-associated hypertension documented. Chronic or unspecified maternal hypertension was documented in 7.4% of deaths^ §§§^ (Figure 2).

**FIGURE 2:**
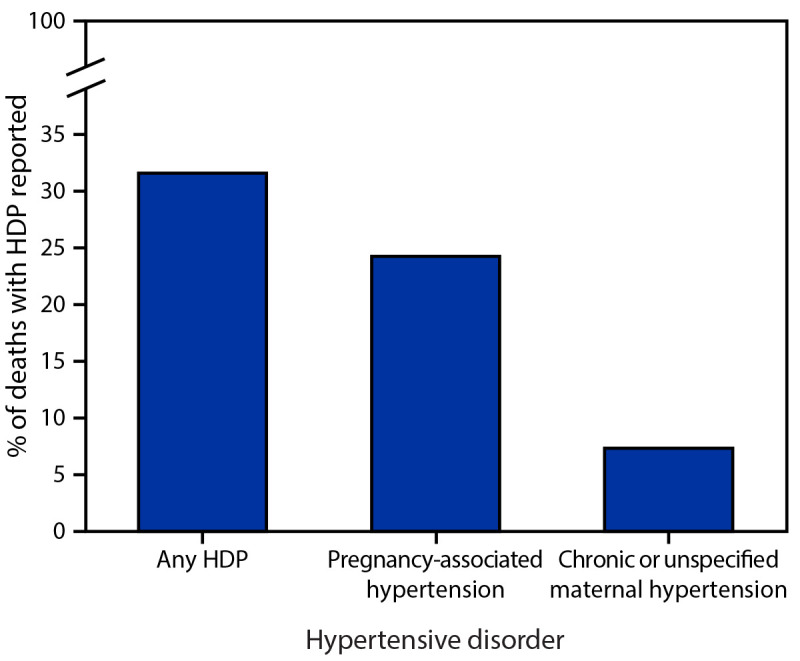
Proportion of deaths* occurring during delivery hospitalization with a documented diagnosis code of a hypertensive disorder in pregnancy^^†^^— National Inpatient Sample, United States, 2017–2019 **Abbreviation:** HDP = hypertensive disorder in pregnancy. * This study did not assign cause of death but instead examined the proportion of in-hospital deaths with an HDP diagnosis code documented among delivery hospitalizations. ^^†^^ HDPs are defined as chronic hypertension, pregnancy-associated hypertension (i.e., gestational hypertension, preeclampsia, eclampsia, and chronic hypertension with superimposed preeclampsia), and unspecified maternal hypertension. Proportions for chronic and unspecified maternal hypertension are combined to conform to the Agency for Healthcare Research and Quality’s data use agreement, which prohibits reporting estimates based on fewer than 11 unweighted observations.

## Discussion

During 2017–2019, HDPs affected approximately one in seven delivery hospitalizations; prevalence increases were largely driven by increases in pregnancy-associated hypertension. HDPs were documented in approximately one in five delivery hospitalizations among Black women and one in three among women aged 45–55 years. An HDP diagnosis code was documented in approximately one in three deaths occurring during delivery hospitalization. Timely diagnosis and treatment of HDP are critical to preventing severe complications and mortality (*1*).

Prevalence of risk factors for HDP, such as advanced maternal age, obesity, and diabetes mellitus, have increased in the United States (*1*), and might explain the increase in HDP prevalence. Women with a history of pregnancy-associated hypertension are at increased risk for cardiovascular disease compared with women with normotensive pregnancies.^¶¶¶ ^Addressing risk factors for HDP across the lifespan is important for preventing HDP and improving future health.****

There are substantial racial and ethnic disparities in HDP prevalence. Compared with non-Hispanic White women, non-Hispanic Black women have higher odds of entering pregnancy with chronic hypertension and developing severe preeclampsia (*3*). Factors that contribute to racial and ethnic inequities in chronic and pregnancy-induced hypertension include higher prevalences of HDP risk factors (*4*), as well as differences in access to health care and the quality of health care delivered (*5*). Racial bias within the U.S. health care system can affect HDP care from screening and diagnosis to treatment (*6*). Furthermore, psychosocial stress from experiencing racism is associated with chronic hypertension (*7*). In a study of racial and ethnic disparities in pregnancy-related deaths, those caused by HDP among Black and AI/AN women were found to be substantially higher than those among White women (*8*), highlighting the importance of addressing HDP to reduce inequities in pregnancy-related mortality.

Regional and rural-urban differences in HDP prevalence have been previously reported (*9*). Place-based disparities in HDP prevalence might be due to differences in prevalence of HDP risk factors, including diet, tobacco use, physical activity patterns, poverty, or access to care.^†††† ^Rural counties are at higher risk for pregnancy-related mortality than metropolitan counties (*10*). A strategy to address place-based disparities in HDP and pregnancy-related mortality can include strengthening regional networks of health care facilities providing risk-appropriate maternal care through telemedicine and transferring delivery care of persons with high-risk conditions to facilities that can provide specialty services.^§§§§^

Clinical guidance for reducing complications from HDP focuses on prompt identification and preventing progression to severe maternal complications. Recommendations for identifying and monitoring pregnant persons with hypertension include measuring blood pressure throughout pregnancy, including self-monitoring.^¶¶¶¶^ Recommendations for preventing preeclampsia include low-dose aspirin for persons at risk and exercise programs.***** Once a diagnosis of an HDP is received, management strategies include blood pressure–lowering medication,^†††††^ prevention of eclamptic seizures (e.g., administration of magnesium sulfate), and close maternal and fetal monitoring and coordination and continuity of care during the postpartum period.^§§§§§^

At the systems level, perinatal quality collaboratives (PQCs)^¶¶¶¶¶^ implement evidence-based quality improvement initiatives in health care facilities, including those to address severe hypertension.****** PQCs use collaborative learning, training, toolkits, and maternal safety bundles (e.g., Alliance for Innovation on Maternal Health Patient Safety Bundles^††††††^) to improve the quality of care and outcomes statewide. Maternal mortality review committees (MMRCs) provide recommendations for preventing future pregnancy-related deaths, including those attributable to HDP, and often collaborate with PQCs to translate MMRC recommendations into clinical and health systems interventions. Health communication campaigns increase awareness of urgent warning signs of HDP that indicate need for immediate care.^§§§§§§^ Strategies to address health inequities in HDP include addressing implicit, institutional, and structural racism, disparate access to clinical care, social determinants of health, and engagement of community partners (*2*).

The findings in this report are subject to at least four limitations. First, identification of delivery hospitalizations and HDP is dependent upon accurate ICD-10-CM coding. Less severe cases of HDP might not be coded. In this study, approximately 4% of HDP was documented as unspecified maternal hypertension, which precludes accurate documentation of HDP type. Second, deaths identified using discharge disposition might underestimate deaths during delivery hospitalization.^¶¶¶¶¶¶^ These data do not represent the universe of pregnancy-related deaths, such as those that occur preceding or after delivery hospitalizations.******* This study did not assign cause of death but instead examined the proportion of in-hospital deaths occurring during delivery hospitalization with an HDP diagnosis code documented. Third, CDC was unable to identify persons who delivered more than once during the study period; the unit of analysis is delivery hospitalization. Finally, small sample sizes did not permit the disaggregation of deaths attributable to less frequent types of HDP and other maternal characteristics.

The prevalence of HDP increased during the 3-year study period with noted racial and ethnic, sociodemographic, and place-based disparities. Severe HDP-associated maternal complications and mortality are preventable with equitable implementation of public health and clinical strategies. These include efforts across the life course for preventing HDP, identifying, monitoring, and appropriately treating those with HDP with continuous and coordinated care, increasing awareness of urgent maternal warning signs, and implementing quality improvement initiatives to address severe hypertension.

**Summary**

**What is already known about this topic?**

Hypertensive disorders in pregnancy (HDPs) are common pregnancy complications and leading causes of pregnancy-related death in the United States.

**What is added by this report?**

During 2017–2019, HDP prevalence among delivery hospitalizations increased from 13.3% to 15.9%. The highest prevalence was among women aged 35–44 (18.0%) and 45–55 years (31.0%), and those who were Black women (20.9%) or American Indian and Alaska Native women (16.4%). Among deaths occurring during delivery hospitalization, 31.6% had a diagnosis code for HDP documented.

**What are the implications for public health practice?**

Severe HDP–associated complications and mortality are preventable with equitable implementation of quality improvement initiatives to recognize and promptly treat HDP and to increase awareness of urgent maternal warning signs.

## Footnotes

*
https://www.cdc.gov/reproductivehealth/maternalinfanthealth/pregnancy-complications-data.htm

†
 https://www.cdc.gov/reproductivehealth/maternal-mortality/pregnancy-mortality-surveillance-system.htm?CDC_AA_refVal=https%3A%2F%2Fwww.cdc.gov%2Freproductivehealth%2Fmaternalinfanthealth%2Fpregnancy-mortality-surveillance-system.htm#causes

§ The U.S. Preventive Services Task Force recommends screening for preeclampsia in pregnant women with blood pressure measurements throughout pregnancy. https://www.uspreventiveservicestaskforce.org/uspstf/recommendation/preeclampsia-screening

¶
https://www.hcup-us.ahrq.gov/db/nation/nis/nisdbdocumentation.jsp

** Delivery hospitalizations were identified using ICD-10-CM diagnosis codes (Z370, Z371, Z372, Z373, Z374, Z3750, Z3751, Z3752, Z3753, Z3754, Z3759, Z3760, Z3761, Z3762, Z3763, Z3764, Z3769, Z377, Z379, O7582, O80, and O82), procedure codes (10D00Z0, 10D00Z1, 10D00Z2, 10D07Z3, 10D07Z4, 10D07Z5, 10D07Z6, 10D07Z7, 10D07Z8, and 10E0XZZ) and diagnosis-related group codes (765, 766, 767, 768, 774, and 775 through September 2018 and 768, 783, 784, 785, 786, 787, 788, 796, 797, 798, 805, 806, and 807 beginning in October 2018). Ectopic and molar pregnancies and pregnancies with abortive outcomes were excluded. CDC excluded an additional 382 delivery hospitalizations that were missing discharge disposition of the patient.

†† Hypertensive disorders in pregnancy were identified using ICD-10-CM diagnosis codes for chronic hypertension (O100, O101, O102, O103, O109, O104, I10, I11, I12, I13, and I15), pregnancy-associated hypertension (chronic hypertension with superimposed preeclampsia [O11], preeclampsia [O14], eclampsia [O15], gestational hypertension [O13]), and unspecified maternal hypertension (O16).

§§ Chronic hypertension is defined as hypertension diagnosed or present before pregnancy or before 20 weeks of gestation. https://www.acog.org/clinical/clinical-guidance/practice-bulletin/articles/2019/01/chronic-hypertension-in-pregnancy

¶¶ Pregnancy-associated hypertension includes gestational hypertension, preeclampsia, eclampsia, and chronic hypertension with superimposed preeclampsia. Gestational hypertension is defined as hypertension occurring after 20 weeks of gestation in persons with previously normal blood pressure. Preeclampsia is defined as gestational hypertension with new-onset proteinuria. In the absence of proteinuria, preeclampsia might be diagnosed in cases of gestational hypertension with new onset thrombocytopenia, renal insufficiency, impaired liver function, pulmonary edema, visual symptoms, or headache unresponsive to medication and not accounted for by alternative diagnoses. Eclampsia is defined as new-onset tonic-clonic, focal, or multifocal seizures in the absence of other causative conditions. Chronic hypertension with superimposed preeclampsia is defined as preeclampsia in women with a history of hypertension before pregnancy or before 20 weeks of gestation. https://www.acog.org/clinical/clinical-guidance/practice-bulletin/articles/2020/06/gestational-hypertension-and-preeclampsia

*** Patient age in years at hospital admission was calculated from the patient birth date and admission date. The Healthcare Cost and Utilization Project (HCUP) classifies race and ethnicity based on separate race and ethnicity data elements. In HCUP’s combined race and ethnicity data element, ethnicity takes precedence over race (i.e., patients with Hispanic ethnicity are classified as Hispanic, and non-Hispanic patients are classified according to their reported race). The HCUP race and ethnicity category Native American is expressed as American Indian and Alaska Native in this report. Payer indicates the expected primary payer and was categorized as private, public (Medicare or Medicaid), or other (self-pay, no charge, or other). Rurality is based on the urban-rural classification of the patient’s county, according to the CDC National Center for Health Statistics urban-rural classification for health care research. Rurality was categorized as metropolitan, micropolitan, and rural (nonmetropolitan and nonmicropolitan). HCUP obtains hospital Census region, as defined by the U.S. Census Bureau, from the American Hospital Association’s Annual Survey of Hospitals.

††† 45 C.F.R. part 46.102(l)(2), 21 C.F.R. part 56; 42 U.S.C. Sect. 241(d); 5 U.S.C. Sect. 552a; 44 U.S.C. Sect. 3501 et seq.

§§§ Proportions for chronic and unspecified maternal hypertension are combined to conform to the Agency for Healthcare Research and Quality’s Data Use Agreement which prohibits reporting estimates based on fewer than 11 unweighted observations.

¶¶¶
https://www.acog.org/clinical/clinical-guidance/practice-bulletin/articles/2019/05/pregnancy-and-heart-disease

****
https://www.thecommunityguide.org/content/tffrs-pregnancy-health-exercise-programs-prevent-gestational-hypertension

††††
https://www.hhs.gov/sites/default/files/call-to-action-maternal-health.pdf

§§§§
https://www.acog.org/clinical/clinical-guidance/obstetric-care-consensus/articles/2019/08/levels-of-maternal-care

¶¶¶¶ The American College of Obstetrics and Gynecology identifies self-monitored blood pressure as a strategy for ongoing monitoring of women with chronic hypertension (https://www.acog.org/clinical/clinical-guidance/practice-bulletin/articles/2019/01/chronic-hypertension-in-pregnancy). CDC’s Million Hearts also promotes self-monitored blood pressure plus clinical support to help persons with hypertension lower their blood pressure. https://millionhearts.hhs.gov/about-million-hearts/optimizing-care/smbp.html?CDC_AA_refVal=https%3A%2F%2Fmillionhearts.hhs.gov%2Ftools-protocols%2Fsmbp.htm

***** https://www.uspreventiveservicestaskforce.org/uspstf/recommendation/low-dose-aspirin-use-for-the-prevention-of-morbidity-and-mortality-from-preeclampsia-preventive-medication; https://www.acog.org/clinical/clinical-guidance/practice-advisory/articles/2021/12/low-dose-aspirin-use-for-the-prevention-of-preeclampsia-and-related-morbidity-and-mortality

†††††
https://www.acog.org/clinical/clinical-guidance/practice-advisory/articles/2022/04/clinical-guidance-for-the-integration-of-the-findings-of-the-chronic-hypertension-and-pregnancy-chap-study

§§§§§
https://www.acog.org/clinical/clinical-guidance/committee-opinion/articles/2018/05/optimizing-postpartum-care

¶¶¶¶¶ https://www.cdc.gov/reproductivehealth/maternalinfanthealth/pqc.htm; https://www.nichq.org/project/national-network-perinatal-quality-collaboratives

****** https://ldh.la.gov/assets/oph/Center-PHCH/Center-PH/maternal/LaPQC/RMMI_Final_Report_LongForm_5-25-2021.pdf; https://gallery.mailchimp.com/244750cf0d942e5d1b1ca3201/files/d82a9758-6b1b-406b-b0bf-7d6cc972783f/ILPQC_HTN_Factsheet_10.17.18.pdf; https://www.michigan.gov/mdhhs/0,5885,7-339--460279--,00.html

††††††
https://safehealthcareforeverywoman.org/council/patient-safety-bundles/maternal-safety-bundles/severe-hypertension-in-pregnancy-aim/
